# Preconception leptin levels and pregnancy outcomes: A prospective cohort study

**DOI:** 10.1002/osp4.399

**Published:** 2020-01-07

**Authors:** Torie C. Plowden, Shvetha M. Zarek, Saima Rafique, Lindsey A. Sjaarda, Enrique F. Schisterman, Robert M. Silver, Edwina H. Yeung, Rose Radin, Stefanie N. Hinkle, Noya Galai, Sunni L. Mumford

**Affiliations:** ^1^ Epidemiology Branch, Division of Intramural Population Health Research Eunice Kennedy Shriver National Institute of Child Health and Human Development Bethesda Maryland USA; ^2^ Program in Reproductive and Adult Endocrinology Eunice Kennedy Shriver National Institute of Child Health and Human Development, National Institutes of Health Bethesda Maryland USA; ^3^ Department of Obstetrics and Gynecology Howard University Hospital Washington DC USA; ^4^ Department of Obstetrics and Gynecology University of Utah Health Sciences Center Salt Lake City Utah USA; ^5^ Department of Statistics University of Haifa Haifa Israel

**Keywords:** leptin, gestational diabetes, pre‐eclampsia, pregnancy outcomes

## Abstract

**Objective:**

Obesity has become a major, worldwide public health issue and is associated with a greater risk of adverse pregnancy outcomes. Leptin, a hormone produced by adipocytes, is elevated in individuals with obesity and may mediate the association between obesity and pregnancy outcomes. Though leptin levels during pregnancy have been associated with pregnancy outcomes, less is understood regarding preconception levels. Therefore, the objective of this study was to evaluate associations between preconception leptin levels and adverse pregnancy outcomes.

**Methods:**

This was a prospective cohort study nested within a large randomized controlled trial conducted at four medical centres in the United States. A total of 1078 women completed the parent study; this analysis involved women who became pregnant during that study (n = 776). Patients were healthy women, ages 18 to 40, attempting to conceive, with 1 to 2 prior pregnancy losses. Participants were followed for less than or equal to 6 cycles while trying to conceive and throughout pregnancy if they conceived. Preconception leptin concentrations were measured in serum collected at baseline then categorized by tertiles (using the lowest as reference group). Weighted log‐binomial regression estimated risk ratios (RR) and 95% confidence intervals (CIs) for pregnancy loss, preterm delivery (PTD), gestational diabetes (GDM), and hypertensive disorders in pregnancy, adjusting for age, waist‐to‐hip ratio (WHR), and body mass index (BMI).

**Results:**

The mean (SD) BMI in this cohort was 25.4 ± 6.0. GDM (RR 18.37; 95% CI, 2.39‐141.55) and hypertensive disorders of pregnancy (RR 2.35; 95% CI, 1.20‐4.61) risks were higher among women in the high tertile after adjusting for age and WHR. The associated risk persisted when adjusting for BMI for GDM but was attenuated for hypertensive disorders in pregnancy. Leptin levels were not associated with risk of pregnancy loss or PTD.

**Conclusions:**

Women with higher baseline preconception leptin levels had a higher likelihood of experiencing some adverse pregnancy outcomes including GDM and hypertensive disorders of pregnancy. These findings warrant further evaluation, especially in light of the association between leptin and obesity.

## 1 INTRODUCTION

Leptin is a hormone produced by adipocytes that regulates energy homeostasis by promoting energy expenditure and inhibiting food intake.^1^ In reproduction, apart from maternal energy metabolism, leptin also plays an important role in fetal growth and development and influences placental functions including implantation, placental angiogenesis, nutrient transport, and immunomodulation.^2^, ^3^ In the setting of obesity, however, leptin resistance ensues, and higher body fat is correlated with circulating levels.^1^, ^4^ As such, the role of leptin while in nonpathological settings may positively signal sufficient resources for reproduction, at higher levels, it may be an indicator of detrimental impact and adipocyte dysfunction.

Given the strong positive association between high leptin levels and body mass index (BMI) and body fat mass,^4^ leptin may mediate the link between the increased risk of pregnancy complications in women with obesity. Leptin has been associated with pre‐eclampsia,^5^, ^6^, ^7^, ^8^, ^9^, ^10^ gestational diabetes (GDM),^11^, ^12^, ^13^ higher rates of neonatal morbidity, mortality, and neonatal ICU admissions in the offspring.^14^, ^15^ However, studies evaluating the relations between leptin and pregnancy outcomes have included only a small number of women and measured leptin levels during pregnancy, which cannot rule out reverse causality. For along with white adipose tissue, the human placenta is a major source of leptin. Leptin levels naturally increase during pregnancy, plateau in the second trimester, and decrease post‐partum.^16^, ^17^ Measurements during pregnancy may have differing levels due to the progression of pregnancy, which could be a consequence of pregnancy complications. Many studies also evaluated specific populations including women undergoing fertility treatment,^3^, ^18^ women with obesity,^19^ or women with a history of three or more pregnancy losses.^20^ Less is known regarding otherwise healthy women. Thus, the goal of this study was to evaluate the associations between preconception leptin levels and adverse pregnancy outcomes in a healthy, fecund population. The hypothesis was that preconception leptin levels would negatively impact pregnancy outcomes.

## 2 METHODS

This was a prospective cohort study from the Effects of Aspirin in Gestation and Reproduction (EAGeR) trial, a multicentre, double‐blind, block‐randomized, placebo‐controlled trial of the effects of low dose aspirin on live birth.^21^, ^22^ Healthy women with a history of one or two previous pregnancy losses were recruited at four clinical sites from 2007 to 2011 (n = 1228). A total of 1078 women completed the parent study; this analysis involved women who became pregnant during that study (n = 776). The detailed study design and methods have been previously published.^21^ Institutional Review Board (IRB) authorization was obtained at all clinical centres and the data coordinating centre. Each participant provided written informed consent and a Data Safety and Monitoring Board (DSMB) monitored patient safety. The trial was registered with http://ClinicalTrials.gov, number NCT00467363.

### 2.1 Study design and population

The study was composed of women aged 18 to 40 years, who were actively attempting to conceive, had regular menstrual cycles (21‐42 days in length), and one or two documented prior pregnancy losses. None of the participants had a known history of infertility and had no prior diagnosis of pelvic inflammatory disease, tubal occlusion, endometriosis, anovulation, uterine abnormality, or polycystic ovarian syndrome.

Study participants were followed throughout pregnancy if they conceived. However, follow‐up was discontinued if women did not become pregnant after 6 cycles of follow‐up. At the preconception baseline visit, participants underwent a physical examination that included measuring height and weight (which were used to calculate BMI), as well as waist and hip circumference (used to calculate waist‐to‐hip ratio [WHR]). Anthropometrics were performed by centrally trained study staff who performed the measurements according to standardized protocols to reduce measurement error.

Preconception leptin concentrations were measured in serum collected at baseline (prior to randomization and use of either placebo or aspirin) which corresponded to days 2 to 4 of each participant's menstrual cycle. The leptin samples were stored at −80°C within 1 hour after collection and were thawed for analysis of leptin in 2014. A subset of participants reported fasting during this visit. The interassay laboratory CVs were 3.3% and 3.2% at mean concentrations of 64.5 and 621 pg mL^−1^ for lyophilized manufacturer's controls and 16.9% for an in‐house pooled serum control (Quantikine ELISA [R&D Systems, Minneapolis, MN]; quantitative sandwich enzyme immunoassay).

### 2.2 Outcome measures

The primary outcomes were pregnancy loss, preterm delivery (PTD), hypertensive disorders of pregnancy, and GDM. Human chorionic gonadotropin (hCG)‐detected pregnancy was determined using hCG measurement on stored urine, clinical urine pregnancy tests, or home pregnancy tests. A clinically confirmed pregnancy was defined as evidence of a continuing intrauterine pregnancy on ultrasound at 6 to 7 weeks of gestation (ie, gestation sac, clinical documentation of fetal heart tones, or a later stage confirmation of pregnancy).

Biochemical pregnancy losses were defined as a positive hCG pregnancy test (utilizing urine or blood) which was followed by absence of signs of clinical pregnancy, with or without missed menses. Clinical pregnancy losses were defined as a pregnancy loss detected after clinical recognition of pregnancy by early ultrasound at approximately 6.5 weeks of gestation.

Gestational age was determined by an ultrasound conducted in early pregnancy (6 to 7 weeks) for 97% of clinically confirmed pregnancies among women who completed the trial; for the remaining 3% of pregnancies, gestational age was determined using menstrual cycle dating from home‐based fertility monitors provided by the study. Pregnancy outcomes, including delivery date, were assessed by post‐partum phone interview and through medical record review by trained EAGeR staff. PTD was defined as a birth occurring between 20 weeks and 0 days and 36 weeks and 6 days of gestation. All cases of PTD were prospectively identified during the study and had further review of abstracted medical records by a maternal‐fetal medicine physician to vet and categorize the outcome as spontaneous, medically indicated, or uncertain. Physicians providing prenatal care to patients made a diagnosis of hypertensive disorders in pregnancy (which included gestational hypertension and pre‐eclampsia) or GDM based on standard clinical and laboratory criteria. During the study interval, a two‐step process with a 1‐hour glucose tolerance screen, followed by a 3‐hour glucose tolerance test was used at all sites.

### 2.3 Statistical analysis

Demographic characteristics were compared by tertile of baseline leptin level using ANOVA or chi‐square tests as appropriate. Weighted log‐binomial regression was used to estimate risk ratios (RR) and 95% confidence intervals (CIs) for pregnancy loss, PTD, GDM, and hypertensive disorders in pregnancy. In some of the models, the number of cases was too low to estimate the RR; in those instances, odds ratios (OR) are reported. Given that pregnancy‐related outcomes are conditional on becoming pregnant, the analysis was restricted to women with an hCG‐detected pregnancy and adjustment for this selection was done using inverse probability weights estimated using factors associated with becoming pregnant (ie, age, parity, marital status, treatment assignment, and number of prior losses).^23^

Unfortunately, there are no established clinical cut‐points for leptin. As such, a data‐driven approach was used and analysis was done using leptin tertiles based on the distribution in the study population. Other studies of leptin have also used a similar tertile approach for categorizing leptin levels.^11^, ^24^ Leptin levels were categorized according to tertiles: low (0.007‐11.3 ng mL^−1^), middle (11.4‐26.2 ng mL^−1^), and high (26.3‐97.4 ng mL^−1^), with the low tertile used as the reference group. Leptin was also evaluated as a continuous variable. All models were adjusted for age and WHR. As leptin was strongly correlated with excess body fat mass,^4^ prior studies have noted that, although widely used as an obesity measure, BMI may not be the best assessment of adiposity.^25^ Accordingly, WHR was proposed as a better measure of adiposity and a better predictor of cardiovascular risk and mortality than BMI^26^, ^27^; thus, results were adjusted for WHR rather than BMI. Sensitivity analyses were performed to assess the effect of additionally adjusting for fasting status, and BMI, as well as to check for potential interactions between WHR and leptin, and potential non‐linearity using restricted cubic splines. All analyses were performed in SAS version 9.4 (SAS Institute, Cary, NC).

## 3 RESULTS

A total of 1228 participants enrolled in the trial, and 776 had an hCG‐detected pregnancy with an available preconception leptin concentration. Pregnant women with available preconception leptin levels who became pregnant were divided into tertiles (n = 776). Fasting was not a requirement for blood collection at the baseline visit; 95 women were fasting, 670 women were not, and 11 women were missing information on fasting status, and mean leptin levels in each tertile were similar between women who were fasting compared with nonfasting (Table 1). Women in each tertile were similar in age, ethnicity, and employment status (Table 1). Women in the high tertile were more likely to have an annual household income of less than $40 000, were less likely to have attained education beyond high school, and were more likely to have a history of pre‐eclampsia. Furthermore, women in the high tertile were more likely to smoke daily and to consume alcohol occasionally. Women had significantly different BMI by tertile (lowest tertile 21.0, middle tertile 24.8, and highest tertile 31.8 kg m^−2^), as well as WHR (lowest tertile 0.78, middle tertile 0.81, and highest tertile 0.83) (Table 1). Women with leptin levels in the highest tertile also had higher blood pressure and lower gestational weight gain.

**Table 1:** Demographics and baseline characteristics by tertile of baseline leptin level: the EAGeR trial

	Total	Leptin Tertile			*P* Value
		Tertile 1: 0.007‐11.3 ng mL^−1^	Tertile 2: 11.4‐26.2 ng mL^−1^	Tertile 3: 26.3‐97.4 ng mL^−1^	
N	776	281	275	220	
Age, y: Mean ± SD	28.7 ± 4.6	29.2 ± 4.6	28.6 ± 4.6	28.2 ± 4.7	.05
BMI, kg m^−2^: Mean ± SD	25.4 ± 6.0	21.0 ± 2.2	24.8 ± 3.6	31.8 ± 6.1	<.0001
WHR: Mean ± SD	0.81 ± 0.07	0.78 ± 0.06	0.81 ± 0.07	0.83 ± 0.07	<.0001
Baseline systolic blood pressure (BP): Mean ± SD	110.9 ± 11.7	107.5 ± 10.3	110.3 ± 11.2	116.1 ± 12.2	<.0001
Baseline diastolic BP: Mean ± SD	71.9 ± 9	70 ± 8.6	71 ± 8.5	75.6 ± 9.2	<.0001
Gestational weight gain: Mean ± SD	12.8 ± 5.5	13.1 ± 4.8	13.5 ± 5.3	11.4 ± 6.4	.003
Fasting leptin: Mean ± SD	27.0 ± 18.5	6.3 ± 3.0	18.6 ± 4.5	43.9 ± 12.8	<.0001
Nonfasting: Mean ± SD	20.8 ± 18.5	6.2 ± 2.7	17.7 ± 4.2	46.3 ± 17.3	<.0001
First degree relative with diabetes: n (%)	161 (21.2)	50 (18.2)	54 (20.1)	57 (26.4)	.080
Prior preterm delivery: n (%)	57 (8.1)	19 (7.4)	21 (8.3)	17 (8.7)	.868
Prior pre‐eclampsia: n (%)	13 (1.9)	4 (1.6)	1 (0.4)	8 (4.1)	.018
White race	769 (96.5)	269 (95.7)	265 (96.4)	214 (97.3)	.66
≤High school^a^	90 (11.3)	23 (8.2)	24 (8.7)	38 (17.3)	.002
Household income (annual)^a^					
≥$100 000	326 (40.9)	106 (37.7)	122 (44.4)	87 (39.5)	.001
$75 000‐$99 999	114 (14.3)	58 (20.6)	37 (13.5)	18 (8.2)	
$40 000‐$74 999	116 (14.6)	43 (15.3)	41 (14.9)	31 (14.1)	
$20 000‐$39 999	187 (23.5)	53 (18.9)	64 (23.3)	62 (28.2)	
≤$19 999	54 (6.8)	21 (7.5)	11 (4)	22 (10)	
Employed^a^	576 (73.4)	196 (71)	205 (75.4)	162 (74.7)	.47
Time from last loss to randomization,^a^ mo					
≤4	476 (60.9)	172 (61.6)	166 (61.7)	123 (57.7)	.85
5‐8	138 (17.6)	51 (18.3)	44 (16.4)	40 (18.8)	
9‐12	51 (6.5)	16 (5.7)	16 (5.9)	18 (8.5)	
>12	117 (15)	40 (14.3)	43 (16)	32 (15)	
Number of previous pregnancies, not including losses					
0	316 (39.6)	98 (34.9)	118 (42.9)	89 (40.5)	.08
1	291 (36.5)	99 (35.2)	107 (38.9)	81 (36.8)	
2	174 (21.8)	76 (27)	46 (16.7)	46 (20.9)	
3	16 (2)	8 (2.8)	4 (1.5)	4 (1.8)	
Number of previous live births					
0	336 (42.2)	105 (37.4)	123 (44.7)	96 (43.6)	.18
1	305 (38.3)	112 (39.9)	109 (39.6)	80 (36.4)	
2	156 (19.6)	64 (22.8)	43 (15.6)	44 (20)	
Smoking in past year^a^					
Never	706 (89.1)	251 (89.3)	245 (90.1)	191 (87.2)	.07
<6 times per week	57 (7.2)	23 (8.2)	20 (7.4)	13 (5.9)	
Daily	29 (3.7)	7 (2.5)	7 (2.6)	15 (6.8)	
Alcohol consumption in past year^a^					
Often	19 (2.4)	13 (4.6)	4 (1.5)	2 (0.9)	.07
Sometimes	238 (30.2)	82 (29.2)	81 (30.3)	67 (30.5)	
Never	532 (67.4)	186 (66.2)	182 (68.2)	151 (68.6)	

*Note.* Values are mean ± SD or n (%) as indicated.

Abbreviations: BMI, body mass index; WHR, waist‐to‐hip ratio.

a Data on were missing for education (n = 1), income (n = 1), employment (n = 41), time from last loss to randomization (n = 18), smoking (n = 10), and alcohol (n = 15).

Women in the middle and high leptin tertiles did not have a higher risk of any pregnancy loss or clinical pregnancy loss compared with the low tertile group (Table 2). Results remained similar after adjusting for age, WHR, or BMI (Table 2). Additional adjustment for fasting status was also completed given the potential effect of a recent meal on leptin levels and similar results were observed (Table 2).

**Table 2:** Association between tertile of preconception leptin and pregnancy loss

	Model	Among Pregnancies	
		Middle vs Low Tertile^a^ RR (95% CI)	High vs Low Tertile^a^ RR (95% CI)
Any pregnancy loss (n = 185)	Model 1^b^	1.09 (0.81‐1.47)	1.14 (0.83‐1.55)
	Model 2^c^	1.17 (0.86‐1.58)	1.26 (0.91‐1.75)
	Model 3^d^	1.08 (0.80‐1.46)	1.13 (0.82‐1.56)
	Model 4^e^	1.10 (0.79‐1.51)	1.10 (0.70‐1.74)
Clinical loss (n = 130)	Model 1^b^	1.14 (0.78‐1.65)	1.10 (0.74‐1.64)
	Model 2^c^	1.20 (0.83‐1.75)	1.20 (0.79‐1.83)
	Model 3^d^	1.09 (0.75‐1.59)	1.06 (0.71‐1.59)
	Model 4^e^	1.15 (0.77‐1.72)	1.10 (0.62‐1.96)

Abbreviations: BMI, body mass index; CI, confidence interval; hCG, human chorionic gonadotropin; RR, risk ratios; WHR, waist‐to‐hip ratio.

a Reference is the low tertile.

b Model 1 is restricted to women who achieved an hCG‐detected pregnancy, with inverse probability weights used control for potential selection bias introduced by restricting to women who achieved pregnancy. Weights were based on factors associated with becoming pregnancy, including age, parity, marital status, number of prior losses, and treatment assignment. Weighted log‐binomial regression was used to estimate risk ratios and 95% confidence intervals.

c Model 2 adjusted for age and WHR.

d Model 3 adjusted for age, WHR, and fasting status.

e Model 4 adjusted for age and BMI.

A higher risk of GDM (RR 18.68; 95% CI, 2.46‐141.44) was observed in women in the high tertile compared with those in the low tertile. This increased risk was persistent after adjusting models for age, BMI, WHR, and fasting status (Table 3). A higher risk of hypertensive disorders in pregnancy (RR 2.42; 95% CI, 1.30‐4.50) also was noted and persisted when adjusting for age, WHR, and fasting status, but this association was attenuated when adjusted for BMI (Table 3). There was no difference in PTD among leptin tertiles in models adjusted for age, WHR, and fasting (Table 3). No significant interactions between WHR and leptin were observed.

**Table 3:** Pregnancy outcomes in relation to preconception leptin

	Model	Among Pregnancies	
		Middle vs Low Tertile^a^ RR (95% CI)	High vs Low Tertile^a^ RR (95% CI)
Preterm delivery (n = 50)	Model 1^b^	1.11 (0.69‐2.09)	1.21 (0.63‐2.33)
	Model 2^c^	1.16 (0.61‐2.20)	1.24 (0.62‐2.49)
	Model 3^d^	1.08 (0.57‐2.03)	1.11 (0.57‐2.16)
	Model 4^e^	1.15 (0.57‐2.32)	1.38 (0.54‐3.54)
Gestational diabetes (n = 22)	Model 1^b^	7.25 (0.89‐58.90)	18.65 (2.46‐141.44)^*^
	Model 2^c^	7.38 (0.91‐59.79)	18.37 (2.39‐141.55)^*^
	Model 3^d^	7.18 (0.88‐58.28)	17.99 (2.36‐137.00)^*^
	Model 4^e^	8.30 (1.01‐68.54)	24.24 (2.67‐219.64)^*^
Hypertensive disorders of pregnancy (n = 56)	Model 1^b^	1.13 (0.56‐2.27)	2.42 (1.30‐4.50)^*^
	Model 2^c^	1.16 (0.57‐2.39)	2.35 (1.20‐4.61)^*^
	Model 3^d^	1.09 (0.54‐2.18)	2.14 (1.15‐3.99)^*^
	Model 4^e^	1.49 (0.47‐2.21)	1.62 (0.69‐4.59)

Abbreviations: BMI, body mass index; CI, confidence interval; hCG, human chorionic gonadotropin; RR, risk ratios; WHR, waist‐to‐hip ratio.

a Reference is the low tertile.

b Model 1 is restricted to women who achieved an hCG‐detected pregnancy, with inverse probability weights used control for potential selection bias introduced by restricting to women who achieved pregnancy. Weights were based on factors associated with becoming pregnancy, including age, parity, marital status, number of prior losses, and treatment assignment. Weighted log‐binomial regression was used to estimate risk ratios and 95% confidence intervals.

c Model 2 adjusted for age and WHR.

d Model 3 adjusted for age, WHR, and fasting status.

e Model 4 adjusted for age and BMI.

When evaluating leptin as a continuous variable, as there was no evidence of non‐linearity using splines, there were no differences in risks of any pregnancy loss, clinical pregnancy loss, or PTD; however, an association with GDM (OR 2.92, 95% CI, 1.58‐5.40 per log unit increase of leptin) and pre‐eclampsia (RR 1.39, 95% CI, 1.01‐1.90 per log unit increase of leptin) was still observed in adjusted models, and there was no evidence of non‐linearity.

## 4 DISCUSSION

Higher preconception leptin concentrations were associated with an increased risk of GDM and hypertensive disorders including pre‐eclampsia, though not with PTD or pregnancy loss. The association between the highest tertile of leptin and hypertensive disorders of pregnancy and GDM persisted even after controlling for age and body size using WHR. These findings warrant further evaluation, especially in light of the obesity epidemic and strong association between leptin levels and obesity, as leptin may hold promise as a marker to predict adverse pregnancy outcomes independent of central adiposity.

The increased risk of pre‐eclampsia and GDM with higher leptin levels are consistent with several other studies.^5^, ^6^, ^7^, ^8^, ^9^, ^10^, ^11^, ^12^, ^13^ In previous studies, leptin levels during pregnancy were observed to be higher among women with pre‐eclampsia compared with controls,^8^, ^9^, ^10^ though no previous studies assessed levels prior to pregnancy and subsequent pre‐eclampsia. Interestingly, women with gene polymorphisms on the leptin receptor gene (LEPR) were also more likely to develop severe pre‐eclampsia.^7^ These results are also consistent with previous studies showing associations between elevated leptin levels during pregnancy and GDM.^11^, ^12^, ^13^ In particular, leptin concentrations in maternal plasma at 13 weeks of gestation were positively associated with GDM risk,^11^ and two recent systematic reviews showed that maternal leptin concentrations in pregnancy were significantly higher in women with GDM versus controls.^12^, ^13^

There is a strong biological rationale to support associations between higher leptin levels and GDM and hypertensive disorders of pregnancy, including pre‐eclampsia, as leptin appears to play a role in angiogenesis in normal early placentation.^10^ Leptin is also expressed by the placenta^16^, ^28^ and may serve to promote healthy placental function by promoting proliferation and survival of trophoblast cells.^3^, ^29^ Poor placental perfusion may increase placental expression of leptin; thus, it is possible that elevated leptin levels act as a marker of placental insufficiency.^10^ The pathogenesis of pre‐eclampsia is not fully understood, but evidence suggests poor trophoblast invasion may play a critical role in the development of the disease.^30^ Women with obesity are also at increased risk of pre‐eclampsia; one hypothesis is that obesity possibly dysregulates leptin function, leading to placental leptin resistance and hyperleptinemia.^10^ A direct impact of leptin on blood pressure and inflammation has also been observed.^10^ Moreover, elevated leptin levels have been implicated in the pathogenesis of insulin resistance.^12^ Production of leptin can be triggered by proinflammatory cytokines; leptin itself also promotes the release of other proinflammatory cytokines, thus creating an environment of chronic inflammation, insulin resistance, and possibly GDM (12).

In this study, no associations between leptin and pregnancy loss or PTD were observed. Though some prior studies have noted an association between leptin and pregnancy loss, these studies were done only in women with obesity^19^ and women with a history of recurrent pregnancy loss or implantation failure^18^, ^20^; thus, results may not be directly comparable. Moreover, leptin levels were assessed during pregnancy which is an important distinction from the present study which assessed leptin during the preconception period.^20^ Determination of preconception leptin and prospective follow‐up strengthened this study's ability to capture pregnancy losses that may have otherwise gone undetected. Notably, null findings in this study for the prospective association between preconception leptin and pregnancy loss does not support the theory that leptin plays a role in early pregnancy loss, which was suggested by prior reports of a correlation between low endometrial expression of leptin and history of implantation failures or spontaneous abortion.^18^, ^20^ The lack of association between leptin and PTD in this study is consistent with a prior study which noted similar leptin levels in pregnant women who ultimately had a PTD compared with those who delivered at term.^31^

There were several strengths in this study including a prospective design, careful assessment of pregnancy loss during early gestation, and data collection by trained personnel. Additionally, prepregnancy WHR was assessed. Although subtle changes in leptin concentrations have been observed over the menstrual cycle,^32^ the levels vary more considerably during pregnancy. Leptin levels are elevated throughout pregnancy, with concentrations increasing even in the first trimester before any observable increase in body weight.^33^ The leptin levels measured in this study were collected prior to pregnancy and were all timed to menstrual cycle phase, reducing cyclical variability and eliminating the variability due to pregnancy. The study population consisted of young, healthy women without a prior diagnosis of infertility or other gynaecologic disorders, and thus, these results may be more generalizable than past studies of more selected populations. Furthermore, the current results were adjusted for WHR, which may be a better marker of central adiposity than BMI and may improve the ability to discern the relations between leptin and outcomes. There were also some limitations. Notably, most of these samples were not collected while the patients were fasting and leptin levels may be affected by recent food intake, although the results remained consistent even after adjusting for fasting status. Additionally, women in the study overall were generally healthy and had a low incidence of poor outcomes. Particularly, there were very low numbers of PTD, which may have limited the power to detect associations, especially in adjusted models. Still, significant associations with hypertensive disorders of pregnancy and GDM were observed despite low numbers of these complications, though the study is unable to separate gestational hypertension from pre‐eclampsia.

In conclusion, higher preconception leptin was not associated with pregnancy loss or PTD. However, higher preconception leptin remained associated with an increased risk of GDM after controlling for either WHR or BMI while the association with hypertensive disorders was noted after controlling for WHR but attenuated when controlling for BMI. Overall, these findings highlight the potential role of preconception leptin levels on adverse pregnancy outcomes. The findings of this study suggest a role of leptin in adverse pregnancy outcomes. The direct clinical implications of these findings are currently unknown, and additional studies are needed to confirm these findings and further explore the utility of leptin as a potential predictor of these outcomes. Understanding the effect of leptin on pregnancy outcomes may help provide insight into why obese women have a higher rate of obstetrical complications. Thus, further study of these factors may help guide counselling, screening, or care recommendations, which is particularly relevant in light of the obesity epidemic plaguing the United States.

## CONFLICT OF INTEREST STATEMENT

The authors declared no conflict of interest.
